# Magic Angle Spinning Solid-State ^13^C Photochemically Induced Dynamic Nuclear Polarization by a Synthetic Donor–Chromophore–Acceptor System at 9.4 T

**DOI:** 10.1021/acs.jpclett.4c01121

**Published:** 2024-05-15

**Authors:** Federico De Biasi, Michael A. Hope, Yunfan Qiu, Paige J. Brown, Máté Visegrádi, Olivier Ouari, Michael R. Wasielewski, Lyndon Emsley

**Affiliations:** † Institut des Sciences et Ingenierie Chimiques, École Polytechnique Fedérale de Lausanne (EPFL), CH-1015 Lausanne, Switzerland; § Department of Chemistry, Center for Molecular Quantum Transduction, Paula M. Trienens Institute for Sustainability and Energy, Northwestern University, Evanston, Illinois 60208-3113, United States; ∥ Aix-Marseille University, Centre National de la Recherche Scientifique (CNRS), Institut de Chimie Radicalaire, 13013 Marseille, France

## Abstract

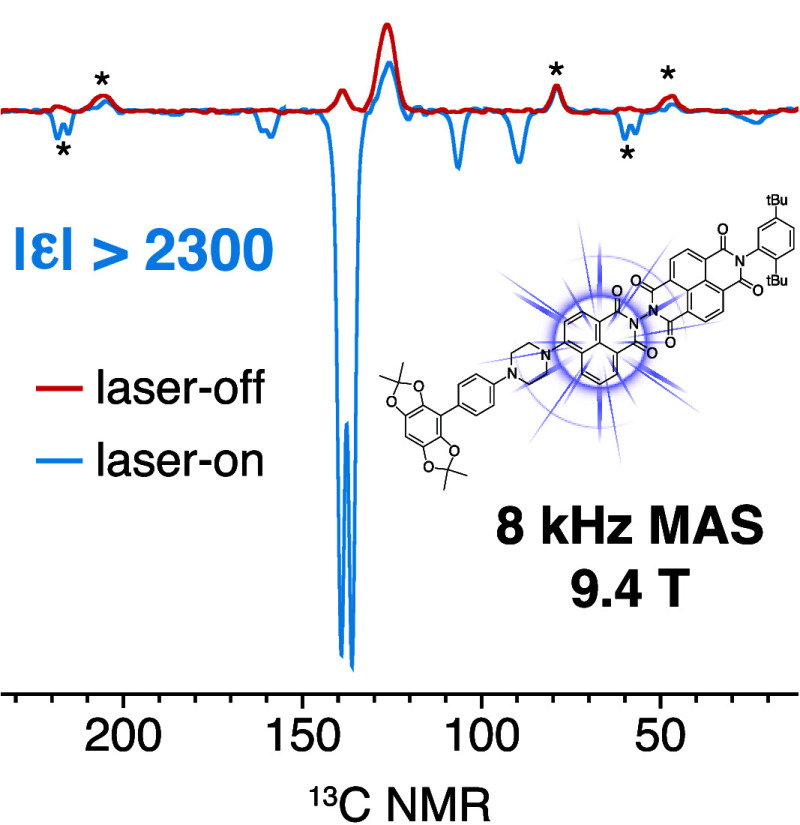

Solid-state photochemically induced dynamic nuclear polarization (photo-CIDNP) is a nuclear magnetic resonance spectroscopy technique in which nuclear spin hyperpolarization is generated upon optical irradiation of an appropriate donor–acceptor system. Until now, solid-state photo-CIDNP at high magnetic fields has been observed only in photosynthetic reaction centers and flavoproteins. In the present work, we show that the effect is not limited to such biomolecular samples, and solid-state ^13^C photo-CIDNP can be observed at 9.4 T under magic angle spinning using a frozen solution of a synthetic molecular system dissolved in an organic solvent. Signal enhancements for the source molecule larger than a factor of 2300 are obtained. In addition, we show that bulk ^13^C hyperpolarization of the solvent can be generated via spontaneous ^13^C–^13^C spin diffusion at natural abundance.

Nuclear magnetic resonance (NMR) spectroscopy is an indispensable tool for chemical and structural analysis owing to its outstanding ability to capture changes in the local environment at the atomic scale. Nonetheless, the flip side of NMR lies in its low sensitivity compared to other spectroscopic methods, where reduced sensitivity is a direct outcome of the small energy gap that separates the nuclear spin states, resulting in only a minor population imbalance, even at liquid nitrogen temperatures (80–100 K).^1,2^

To combat the low sensitivity of NMR, several methods have been designed to enhance the spin population imbalance beyond the thermal equilibrium, generating so-called hyperpolarization.^3−9^ These hyperpolarization methods in NMR spectroscopy boost the sensitivity of the technique by orders of magnitude, allowing complex analyses to be performed, which would otherwise not be possible.^3,10^ In the solid state, the most widespread NMR hyperpolarization protocol is dynamic nuclear polarization (DNP),^11,12^ where, upon microwave irradiation, thermal electron spin polarization is transferred from a paramagnetic species to neighboring nuclei, typically ^1^H spins, and then is relayed to the bulk of the sample by spontaneous spin diffusion.^13,14^

The signal enhancement (ε) provided by DNP is limited, among other factors, by the magnitude of the thermal electron spin polarization and cannot theoretically exceed a factor of 658 for ^1^H nuclei or 2610 for ^13^C nuclei (ε_DNP_^max^ = |γ_e_/γ_N_|, where γ_e_ and γ_N_ are the electron and nuclear gyromagnetic ratios).^2^ On the other hand, optically induced NMR hyperpolarization methods exploit transient excited states as the initial source of polarization. Such excited states can be characterized by a non-Boltzmann spin population imbalance; therefore, they can provide nuclear hyperpolarization exceeding the DNP limit, ε_DNP_^max^.^15−20^

One optical NMR hyperpolarization protocol is photochemically induced dynamic nuclear polarization (photo-CIDNP).^21−25^ In the solid state, photo-CIDNP is observed in donor–acceptor systems that undergo charge separation upon irradiation with light, generating a transient spin-correlated radical pair (SCRP).^26^ The SCRP is typically generated in the singlet state, |S⟩. At high fields, the SCRP coherently evolves between the singlet and the |T_0_⟩ triplet state, potentially accumulating nuclear hyperpolarization during the process.^22,27,28^ Until now, solid-state photo-CIDNP at magnetic fields larger than 1 T has been investigated only in flavoproteins and photosynthetic reaction centers, where solely ^13^C and ^15^N nuclei have been directly polarized.^26,29−44^ Recently, we reported the first example of direct ^1^H photo-CIDNP in solids, at 0.3 T, using a synthetic donor–chromophore–acceptor (D–C–A) molecular system dissolved in a glassy frozen matrix.^45^ In this study, ^1^H–^1^H spin diffusion was exploited to relay the polarization from the D–C–A system to the glassy matrix, leading to bulk uniform polarization of the sample. This was in contrast with most solid-state photo-CIDNP studies on photosensitive proteins, where the poor spin diffusion efficiency of ^13^C and ^15^N nuclei at natural abundance confines the polarization in the vicinity of the donor–acceptor system.

Here, we extend the concept of solid-state photo-CIDNP hyperpolarization by a D–C–A molecule to the high magnetic fields required for high-resolution NMR (here, 9.4 T). The advantages of small molecules over biomolecular systems, such as flavoproteins and photosynthetic reaction centers, are the easier tuneability of the photoactive machinery and broader solvent compatibility. However, to the best of our knowledge, no solid-state photo-CIDNP activity has been reported on D–C–A molecular systems at fields >1 T.

In the present work, we show that solid-state ^13^C photo-CIDNP can occur in a D–C–A molecule in a frozen solution at 9.4 T, where D is benzobisdioxole aniline (BDX), C is 4-aminonaphthalene-1,8-dicarboximide (ANI), and A is naphthalene-1,8:4,5-bis(dicarboximide) (NDI). We refer to this motif as CarboPol. The structure of CarboPol is shown in Figure 1, together with its photocycle. Upon absorption of a photon by the chromophore, an excited state D–C*–A is populated, which then undergoes an intramolecular electron transfer to form a charge-separated state. This charge-separated state is a singlet-born SCRP, indicated as ^1^(D^+^ ^•^–C–A^–^ ^•^) in Figure 1. The radical pair then evolves between that and the |T_0_⟩ state, denoted as ^3^(D^+^ ^•^–C–A^–^ ^•^), and eventually decays to either the initial state or the neutral triplet state via charge recombination (CR). In the case of CarboPol, the neutral triplet that is created after CR in the triplet channel (^3^CR) is localized on the acceptor and has the form D–C–^3^A.^46^

**Figure 1:**
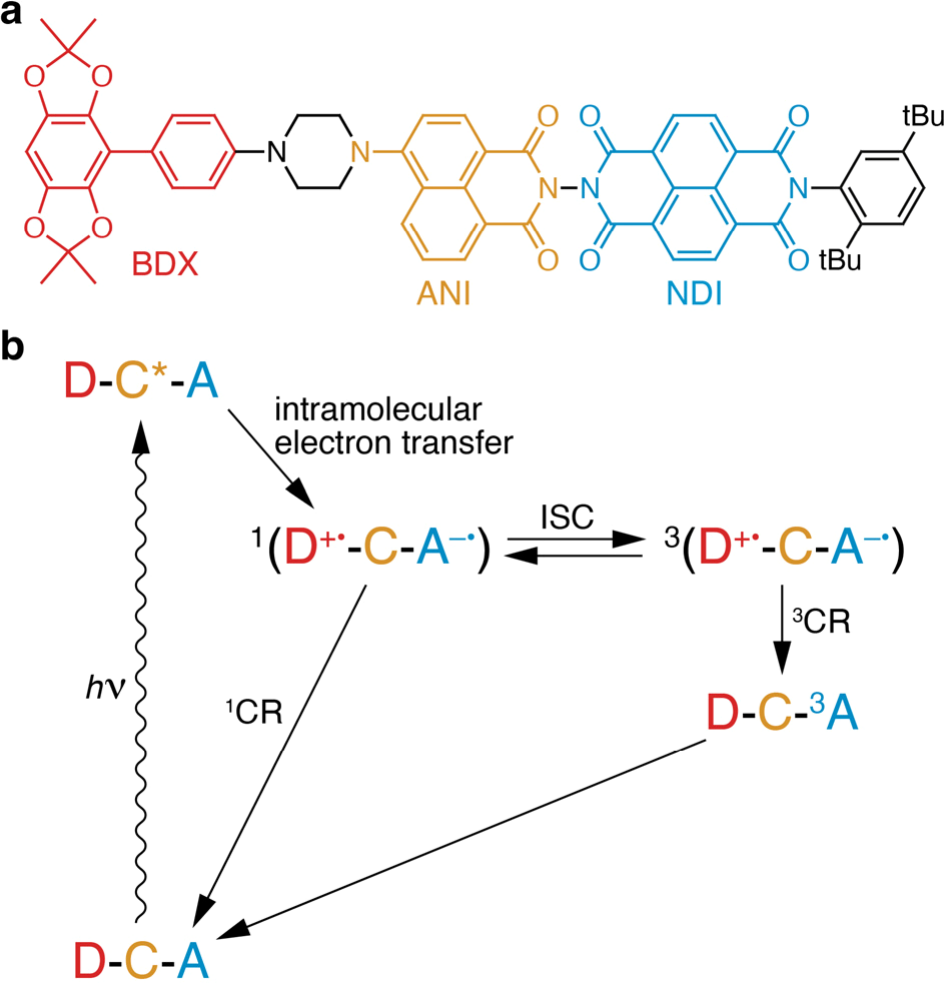
(a) Structure of the CarboPol molecule introduced in this work. The donor (BDX), chromophore (ANI), and acceptor (NDI) in the photoactive part of the structure are shown in red, yellow, and blue, respectively. The linker and end group are shown in black. (b) Photocycle of the D–C–A system in CarboPol (*h*ν, photoexcitation; ISC, intersystem crossing; and CR, charge recombination).

CarboPol was synthesized as detailed in the Supporting Information. The molecule was designed on the basis of an established family of very well-characterized D–C–A molecules.^46−56^ It shares features with the previously introduced PhotoPol molecule that showed a ^1^H photo-CIDNP response at 0.3 T.^45^ CarboPol was designed to have a stronger electron–electron coupling as a result of the shorter donor–acceptor distance.

For all of the NMR experiments presented here, *o*-terphenyl (OTP) was used as a glassy matrix for its favorable solvation and glass-forming properties, optical transparency, and long nuclear spin longitudinal relaxation times.^57−60^^13^C photo-CIDNP experiments were carried out at 9.4 T (100.6 MHz ^13^C Larmor frequency) and cryogenic temperatures (100–220 K) using an Avance III HD Bruker NMR spectrometer and a commercial low-temperature magic angle spinning (MAS) DNP probe, in which a portion of the microwave waveguide was modified to allow for optical irradiation of the spinning sample (Figures S1–S3 of the Supporting Information). Samples were packed in a 3.2 mm sapphire rotor to allow for light penetration and sealed with a silicone plug. The MAS rate was 8 kHz in all of the experiments. Sample irradiation was achieved with a continuous wave 450 nm blue laser (the laser output power is 1.3 W, but the power delivered to the sample is likely to be significantly lower). In the laser-on experiments, the laser was on for the whole duration of the experiments. Experiments at 0.3 T were performed as described previously,^45^ using a laser intensity of 4.2 W cm^–2^.

First, we note that CarboPol shows solid-state ^1^H photo-CIDNP activity at 0.3 T (Figure S13 of the Supporting Information), giving a bulk ^1^H signal enhancement factor of ε_1H_^bulk^ = −27 at a 20 s recycle delay (ε = *I*_on_/*I*_off_). This bulk enhancement factor is 1.7 times larger than that previously observed with PhotoPol under similar conditions. CarboPol has a larger electron–electron coupling compared to that of PhotoPol (*d* ≈ −60 MHz versus −5.5 MHz), as measured in butyronitrile at 85 K (details in the Supporting Information). This larger coupling could enable three-spin mixing at higher fields.^27,28^

Figure 2 shows the direct excitation ^13^C NMR spectra of a 1.5 mM frozen solution of CarboPol in OTP at 9.4 T and 100 K with (blue) and without (red) 450 nm laser irradiation, using a recycle delay of 1.8 s. In the absence of irradiation, only the OTP solvent signal is detected. With light irradiation, the ^13^C signals of the CarboPol molecule can clearly be observed. Interestingly, all of the ^13^C photo-CIDNP signals are negatively enhanced, irrespective of whether they belong to the donor or acceptor part of the photoactive machinery. A tentative assignment is also shown based on ^13^C chemical shifts and solution-state NMR data (Figures S4–S7 of the Supporting Information). Note that the large signal from the silicone plug is due to its short ^13^C *T*_1_ compared to OTP, given the short experimental recycle delay. The reduction of the OTP signal at 126 ppm in the laser-on spectrum could be due to an overlap with negatively enhanced ^13^C signals of the NDI acceptor (Figure S5 of the Supporting Information).

**Figure 2:**
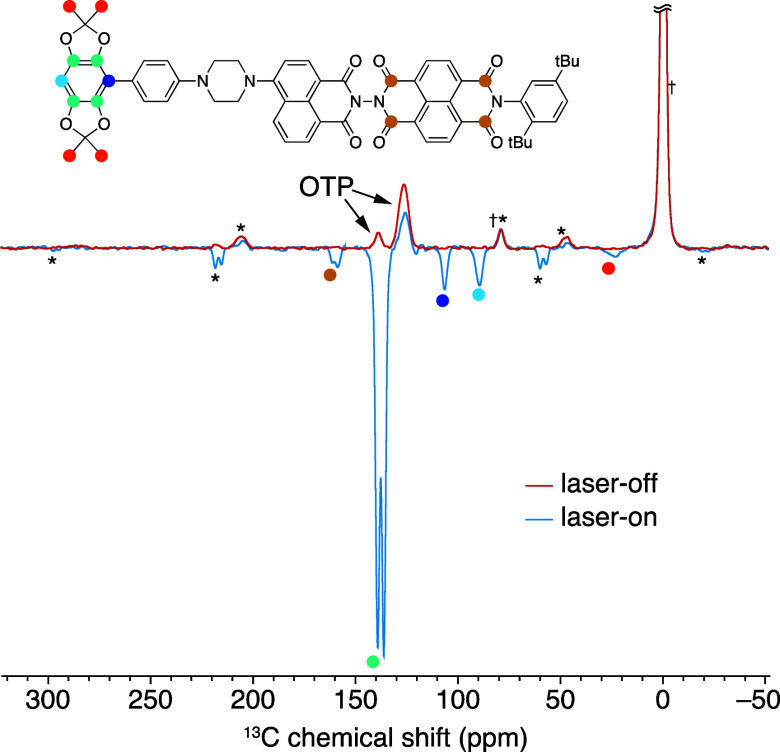
^13^C NMR spectra of a 1.5 mM frozen solution of CarboPol in OTP (9.4 T, 8 kHz MAS, and 100 K) without (red) and with (blue) continuous wave 450 nm laser irradiation. The recycle delay (τ) was set equal to the polarization build-up time under laser irradiation (*T*_b_ = 1.8 s; data shown below), and an excitation pulse of 68° was used to maximize the signal-to-noise ratio per unit time in the laser-on spectrum (Ernst angle for τ = *T*_b_).^61^ Both spectra were acquired with 30 000 scans. The asterisks indicate spinning sidebands, while the daggers denote the silicone plug signals. The colored circles on the spectrum and on the molecular structure shown in the inset indicate the assignment of the photo-CIDNP-enhanced signals (the complete assignment in solution is shown in the Supporting Information).

Because the CarboPol signal without irradiation is too weak to be observed at 1.5 mM with 30 000 scans (15 h), it is not possible to determine the enhancement factor directly. Using the noise level in the laser-off spectrum, we can obtain a lower bound for the magnitude of the ^13^C enhancement factor for the signals at 138 ppm (corresponding to four of the aromatic sites in the BDX donor) of |ε_13C_| > |−340| = 340 at a 1.8 s recycle delay (ε = *I*_on_/*N*_off_, with *I* and *N* being the signal intensity and the noise level). To obtain a higher sensitivity laser-off spectrum, we repeated the same laser-off experiment on two separate 15 mM CarboPol samples in OTP, i.e., 10 times more concentrated than the sample used to acquire the spectra in Figure 2. Further details are given in the Supporting Information. Even at 15 mM, no CarboPol aromatic signals were observed without laser irradiation after 30 000 scans (Figures S8–S10 of the Supporting Information). This led to an improved estimate of the enhancement factor of the signals at 138 ppm at a 1.8 s recycle delays of |ε_13C_| > 2300.

Degradation of the 1.5 mM CarboPol sample was also investigated during a 10 h period of continuous 450 nm laser irradiation at 100 K. Figure 3 reports the normalized integrated intensity of the BDX donor aromatic signals at 138 ppm as a function of time. After 10 h, the peak area was reduced only by 3%, suggesting that degradation of the sample as a result of laser irradiation is very slow under MAS at this temperature. Therefore, long experiments on this and similar systems are, in principle, feasible, without significant loss in quality over time of the recorded NMR signal.

**Figure 3:**
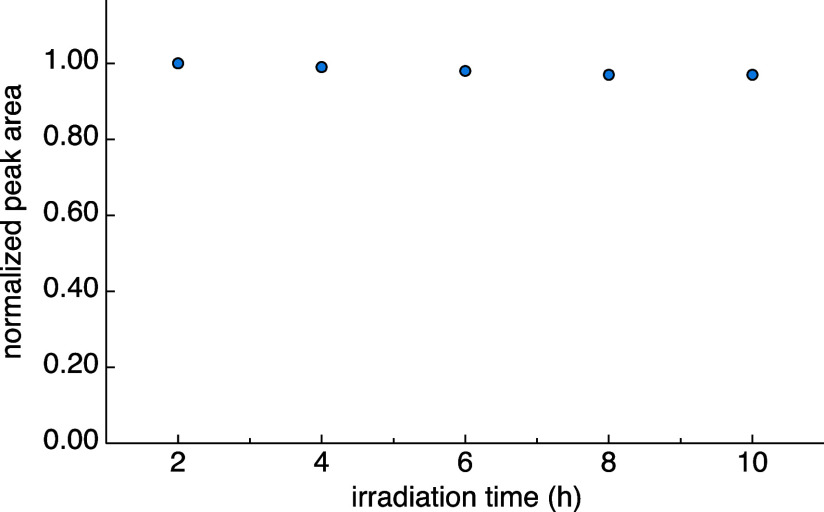
Intensity of the photo-CIDNP effect in CarboPol as a function of the irradiation time with a continuous wave 450 nm laser. Each data point corresponds to the area of the BDX donor aromatic peak at 138 ppm in a direct excitation NMR experiment, each acquired over a 2 h period using an interscan delay of 1.8 s and an excitation pulse of 68° (3972 scans per data point). Data are normalized by the signal area in the spectrum after the first 2 h. Error bars are smaller than the symbol size.

The effect of the temperature on the ^13^C photo-CIDNP activity in CarboPol has also been investigated with a 1.8 s recycle delay within the 100–220 K range, below the glass transition temperature of OTP (243 K).^57^ The magnitude of the ^13^C photo-CIDNP-enhanced signals decreases with an increasing temperature, as shown in Figure 4. The effect is likely due to a shorter SCRP lifetime at higher temperatures, but other parameters (e.g., the electron–electron coupling *d*) might depend upon the temperature as well, affecting the photo-CIDNP activity. The increasing magnitude of the OTP signal at 126 ppm with the temperature is ascribed to either faster ^13^C *T*_1_ relaxation for OTP at higher temperatures or a reduction of the negative photo-CIDNP effect for the CarboPol resonances underneath the OTP signal (especially those from the carbons of the NDI acceptor; Figure S5 of the Supporting Information) or to a combination of the two effects. No sample degradation occurred during the acquisition of the spectra in Figure 4, as suggested by the full recovery of the photo-CIDNP effect when the sample was cooled down again to 100 K (Figure S11 of the Supporting Information).

**Figure 4:**
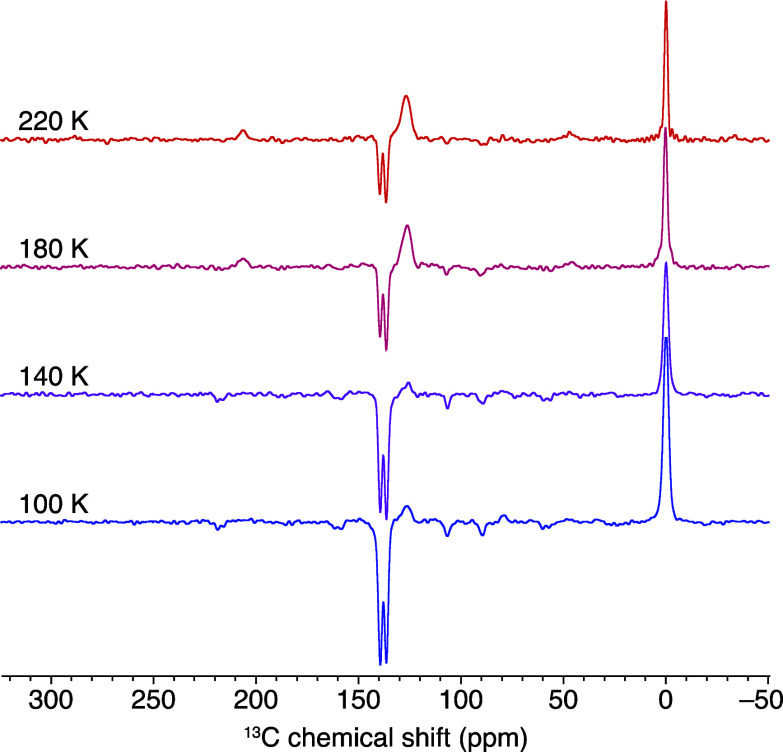
^13^C NMR spectra of a 1.5 mM frozen solution of CarboPol in OTP (9.4 T, 8 kHz MAS) in the presence of 450 nm laser irradiation. Spectra were collected at the indicated temperatures from colder to warmer using a 1.8 s recycle delay, 2000 scans, and an excitation pulse of 68°.

The polarization build-up with laser irradiation of the CarboPol signals at 138 ppm was characterized on the same sample (1.5 mM CarboPol in OTP) at 100 K and 8 kHz MAS. Results are shown in Figure 5. Data were fit to a biexponential function *I*(*t*) = *I*_fast_(1 – e^–*t*/*T*_b,fast_^) + *I*_slow_(1 – e^–*t*/*T*_b,slow_^), where the fast component corresponds to the BDX donor aromatic signals and the slow component corresponds to the overlapping OTP signal. The characteristic build-up time of the photo-CIDNP-enhanced signal was estimated to be 1.8 s. The characteristic build-up time of OTP was not accurately determined here as a result of the long experimental time that would be required.

**Figure 5:**
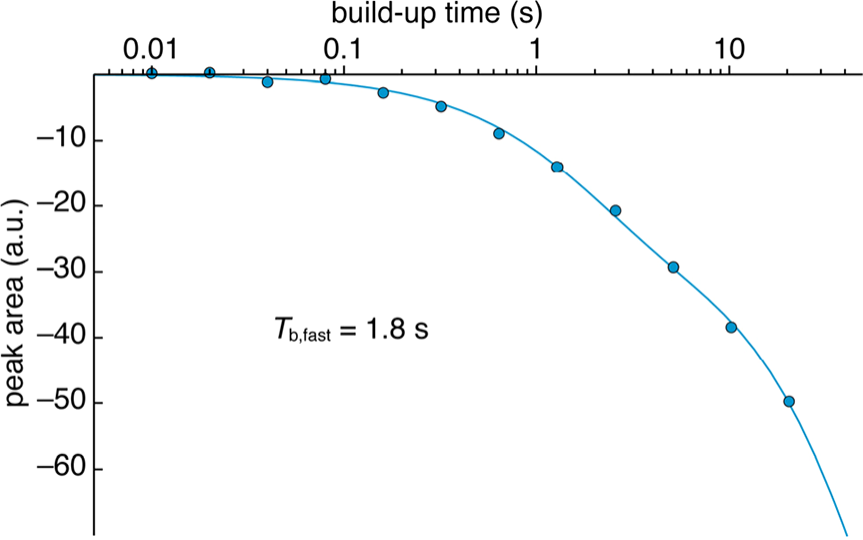
Polarization build-up at 100 K in the presence of continuous wave 450 nm laser irradiation of the CarboPol aromatic signals at 138 ppm (from the BDX donor). The solid blue line shows the best fit to a biexponential function (see the main text for details). Error bars for each data point have a comparable size to the symbols and have been omitted.

From the build-up curve in Figure 5, it is evident that, for longer recycle delays, the overlapping OTP solvent signal also becomes negatively enhanced, as the overall integrated intensity of the signals at 138 ppm continues to decrease even at times longer than 5*T*_b,fast_. This can be seen directly from the other OTP peak at 126 ppm, which becomes negative in the laser-on spectrum with a recycle delay of 20 s (Figure S12 of the Supporting Information). This evidence demonstrates that the ^13^C polarization propagates from the CarboPol molecules to the OTP solvent molecules in the bulk of the sample via spontaneous ^13^C–^13^C spin diffusion, despite the very low efficiency of the process (all compounds here are at natural ^13^C abundance). Indeed, ^13^C–^13^C spin diffusion can still occur because of the very long ^13^C *T*_1_ of OTP, which is expected to be on the order of 1000 s at 100 K. Assuming that the spin diffusion coefficient (*D*) is proportional to γ_N_^2^*c*^1/3^,^62^ where *c* is the concentration of the nuclear spins (0.86 M for ^13^C in pure OTP), we can estimate its value for ^13^C spins in bulk OTP to be *D* = 11 nm^2^ s^–1^ by rescaling the measured spin diffusion coefficient of ^19^F in CaF_2_ || [001].^62^ Considering a ^13^C *T*_1_ of 1000 s, the ^13^C spin diffusion length, defined as λ = , is approximatively 100 nm. This analysis does not account for the influence of MAS on *D*,^63^ which could significantly reduce the value of λ for dilute and low-γ spins because of the reduced homonuclear dipolar interaction. Nevertheless, to explore the extent of bulk ^13^C polarization that can be generated at 100 K via ^13^C–^13^C spin diffusion from the CarboPol molecules in this formulation, we measured two spectra with a long recycle delay of 2 h (Figure 6). Without light (red), conventional *T*_1_ relaxation restores the OTP polarization to its thermal equilibrium, while in the presence of light (blue), the large negative ^13^C polarization is relayed via spin diffusion from CarboPol, which now effectively acts as a polarizing agent, to the bulk of the sample. The ^13^C bulk enhancement at a 2 h recycle delay under 8 kHz MAS is ε_13C_^bulk^ = −4.7 (ε = *I*_on_/*I*_off_). Note that the signal from the silicone plug is not enhanced and, with this long recycle delay, is now dwarfed by the OTP signal. The large difference between the high local enhancements on CarboPol and the modest OTP bulk enhancement is ascribed to the very limited ^13^C–^13^C spin diffusion efficiency.^64^

**Figure 6:**
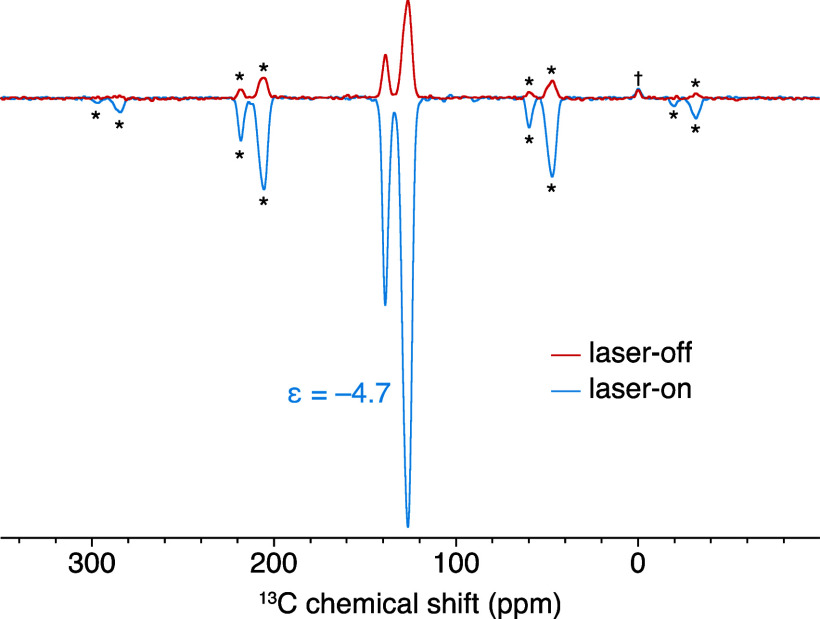
^13^C NMR spectra of a 1.5 mM frozen solution of CarboPol in OTP (9.4 T, 8 kHz MAS, and 100 K) without (red) and with (blue) laser irradiation (2 h recycle delay and 90° excitation pulse). Both spectra were acquired with four scans. The asterisks indicate spinning sidebands, while the dagger denotes the silicone plug signal.

We now consider the photo-CIDNP mechanism at play here. The simplest Hamiltonian used to describe high-field photo-CIDNP of a single nuclear spin can be expressed as^28^
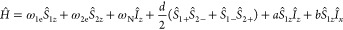
where ω_1e_ and ω_2e_ are the electron spin Larmor frequencies of the SCRP, ω_N_ is the nuclear spin Larmor frequency, *d* is the electron–electron interaction strength, and *a* and *b* are the secular and pseudo-secular components of the hyperfine interaction (coupling with only one electron of the SCRP is assumed). Solid-state photo-CIDNP generates nuclear hyperpolarization according to three different mechanisms: differential relaxation (DR),^30,65^ differential decay (DD),^41^ and three-spin mixing (TSM).^66,67^ These mechanisms are not mutually exclusive and could act simultaneously;^27,28^ however, the values of the spin interactions in CarboPol and the sign of the photo-CIDNP enhancement allow us to determine which mechanism dominates.

DR is most efficient when |Δω_e_| = |ω_1e_ – ω_2e_| = |*a*/2|, while DD is most efficient when |ω_N_| = |*a*/2|.^30,41^ TSM can occur under two different regimes: a first regime involving a double matching condition of the form |Δω_e_| = |ω_N_| = |*a*/2| and a second regime occurring when |ω_N_| = .^27,28,66−68^

On the basis of the calculated *g*-factors of the BDX and NDI cationic and anionic radicals in CarboPol,^46^ |Δω_e_| at 9.4 T is expected to be between 100 and 300 MHz for most molecular orientations. At the same time, |ω_N_| for ^13^C nuclei at 9.4 T is 100.6 MHz. Therefore, for maximum efficiency, both DR and DD require hyperfine couplings on the ^13^C sites in CarboPol on the order of a few hundred MHz, which is unrealistic for this system.^46^ On the other hand, the relative sizes of ω_N_ and *d* (ω_N_ = −100.6 MHz and *d* ≈ – 60 MHz; details in the Supporting Information) suggest that TSM can still occur in a regime for which the matching condition is approximatively satisfied (|ω_N_| ≈ |*d*|). Indeed, we designed CarboPol to have a short donor–acceptor distance for which the electron–electron coupling is dominated by a scalar interaction on the order of 100 MHz. The sign of the signal enhancement via the TSM mechanism only depends upon the sign of *d* and is expected to be negative for both the donor and acceptor sites, which is indeed the case. This also adds weight to the hypothesis that TSM dominates over DR and DD, as the acceptor signal would dominate for DR, while for DD, both positive and negative enhancements would be expected.^28,30,41,46,65^

In conclusion, we have shown that solid-state ^13^C photo-CIDNP can be observed in synthetic donor–acceptor systems at a high magnetic field, if the electronic properties can be designed to match the photo-CIDNP conditions. The effect was proven to be active at 9.4 T and up to 220 K under MAS at 8 kHz, yielding ^13^C enhancements in CarboPol of |ε_13C_| > 2300 at 100 K. Owing to the long ^13^C *T*_1_ of the glassy OTP matrix, spontaneous ^13^C–^13^C spin diffusion can relay the ^13^C photo-CIDNP polarization far from the optically active molecules, resulting in bulk ^13^C hyperpolarization, despite the low efficiency of spin diffusion for ^13^C spins at natural abundance. In the system investigated here, we determined that ^13^C photo-CIDNP is most likely mediated by the three-spin mixing mechanism, for which net polarization is generated.^28,66−68^ These results represent an important step toward generalized signal enhancements in high-field NMR spectroscopy by optical hyperpolarization using photo-CIDNP of tailorable donor–acceptor molecular systems.

## Supporting Information Available

Synthetic methods and characterization of CarboPol, diagrams and pictures of the probe modifications used here, supplementary figures and tables, and a link to all of the raw NMR data (PDF).
