# Supplementary material for: Magic Angle Spinning Solid-State 13C Photochemically Induced Dynamic Nuclear Polarization by a Synthetic Donor–Chromophore–Acceptor System at 9.4 T
Source: J Phys Chem Lett. 2024 May 15;15(20):5488–94. doi: 10.1021/acs.jpclett.4c01121 (PMC11129313; doi:10.1021/acs.jpclett.4c01121)
Supplement: Supplementary file 1 — jz4c01121_si_001.pdf [file jz4c01121_si_001.pdf]

Supporting information

Magic Angle Spinning Solid-State ^{13}C Photo-CIDNP by a Synthetic Donor–Chromophore–Acceptor System at 9.4 T

Federico De Biasi,[1] Michael A. Hope,[1,‡] Yunfan Qiu,[2] Paige J. Brown,[2] Máté Visegrádi,[1] Olivier Ouari,[3] Michael R. Wasielewski,[2] and Lyndon Emsley[1,*]

[1] *Institut des Sciences et Ingenierie Chimiques, École Polytechnique Fedérale de Lausanne (EPFL), CH-1015 Lausanne, Switzerland*

[2] *Department of Chemistry, Center for Molecular Quantum Transduction, Paula M. Trienens Institute for Sustainability and Energy, Northwestern University, Evanston, IL 60208-3113, USA*

[3] *Aix-Marseille University, CNRS, Institut de Chimie Radicalaire, 13013 Marseille, France*

Raw NMR Data

All the raw NMR data associated with the manuscript can be accessed at the following link DOI: 10.5281/zenodo.11184222 and is available under the CC-BY-4.0 (Creative Commons Attribution-ShareAlike 4.0 International) license.

Summary

1) Probe modification for sample irradiation with light under MAS....................S2
2) Solution-state NMR data....................S3
3) Experiments on 15 mM CarboPol samples....................S5
4) Additional solid-state NMR experiments....................S7
5) Electron–electron interaction in CarboPol....................S8
6) Synthetic procedures....................S8

1) Probe modification for sample irradiation with light under MAS

A Bruker 400 MHz 3.2 mm MAS DNP probe has been modified to allow for optical irradiation of the sample under magic angle spinning. All optical parts were purchased from ThorLabs GmbH. The microwave mirrors of the double miter bend on top of the microwave waveguide in the probe were replaced with protected silver mirrors (Ø7.0 mm) mounted in a custom brass piece (Fig. S1).The bottom part of the waveguide that protrudes below the probe base was connected to a custom made apparatus (Fig. S2) that collimates the diverging beam exiting from an optical fiber and directs it into the hollow microwave waveguide in the probe. Fig. S3 shows a closeup of the custom made aluminum adapter used to connect the apparatus in Fig. S2 with the probe waveguide. Two 45° adjustable mirrors (Fig. S2) are used to align the laser with the sample. When properly aligned, it was found that 450 nm light triggers the MAS reading sensor of the probe if no rotor is present in the stator. The alignment procedure consists of adjusting the position of the two 45° mirrors (with no sample in the probe) to maximize the value detected by the MAS II unit (typically within 35,000 and 42,000 at 100 K, with 600 mbar each of cold bearing and drive gas).

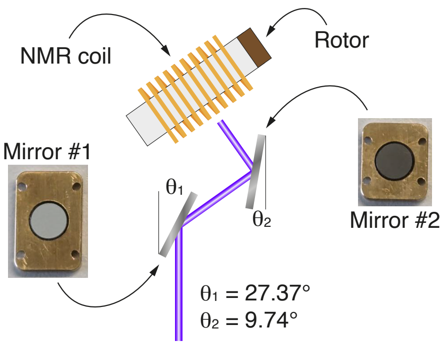

Figure S1. Schematic representation of optical path followed by the light beam in the probe waveguide. The microwave mirrors on the double miter bend are replaced with optical mirrors. The NMR sample is irradiated radially.

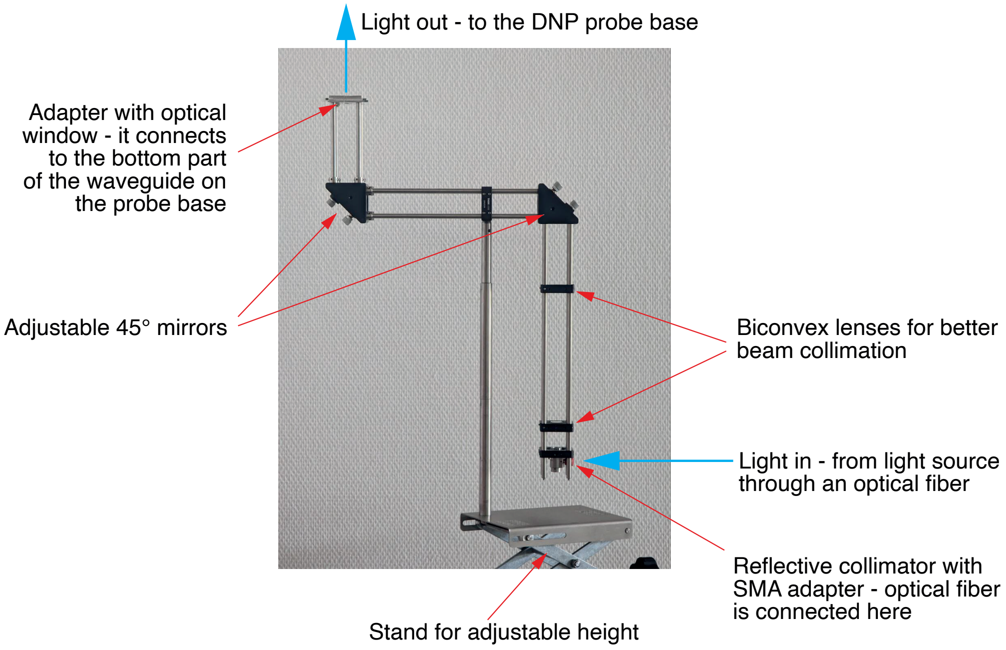

Figure S2. Picture of the apparatus to collimate the beam from optical fiber and direct it in the probe waveguide. During operation, the beam path is entirely shielded with plastic covers.

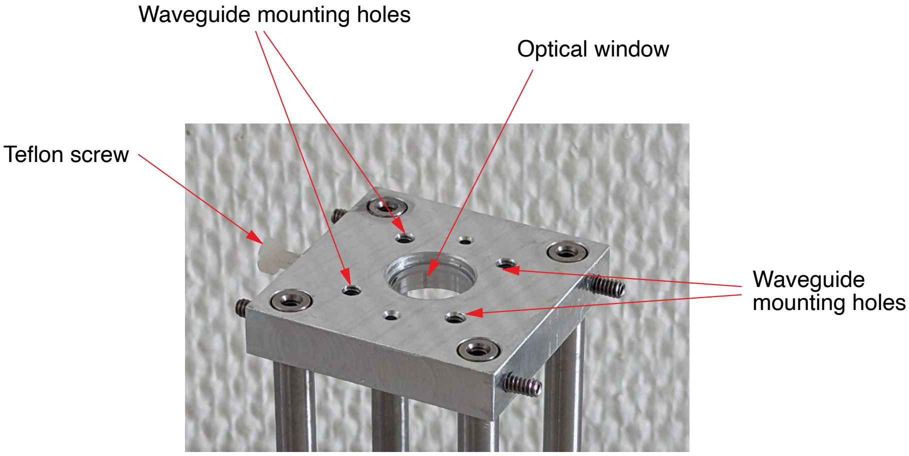

Figure S3. Closeup of the custom-made aluminum adapter used to connect the system in Fig. S2 to the probe microwave waveguide.

2) Solution-state data

The resonances of CarboPol in ^{13}C solid-state NMR spectra were assigned according to solution-state NMR experiments, namely, quantitative ^{1}H NMR (Fig. S4), ^{1}H-^{13}C HSQC and HMBC (Fig. S5), ^{1}H–^{1}H NOESY (Fig. S6) and ^{1}H–^{1}H TOCSY (Fig. S7). All solution-state experiments were performed at 11.7 T and 298 K on a 5 mM CarboPol sample in acetone-d_6 using an Avance Neo Bruker NMR spectrometer equipped with a cryoprobe.

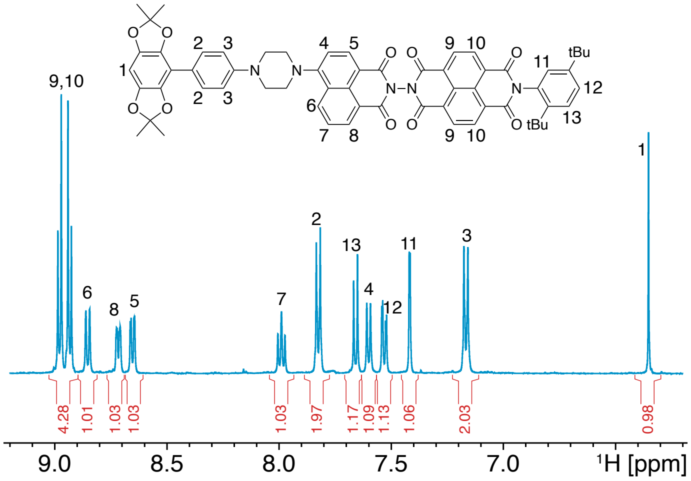

Figure S4. Quantitative ^{1}H NMR spectrum of 5 mM CarboPol in acetone-d_6 (500 MHz ^{1}H Larmor frequency). Only the aromatic region is shown for clarity. The relative integrals are reported in red below each resonance. The full assignment of the ^{1}H aromatic resonances is also reported.

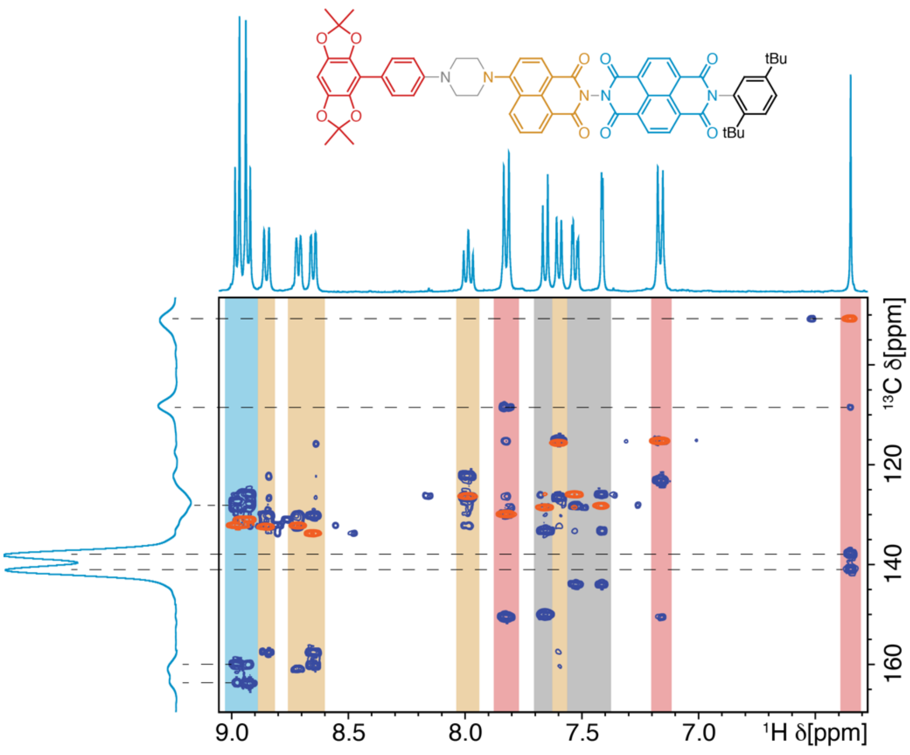

Figure S5. ^{1}H-^{13}C HSQC (orange) and HMBC (dark blue) spectra of 5 mM CarboPol in acetone-d_6 (500 MHz ^{1}H Larmor frequency, 125 MHz ^{13}C Larmor frequency). Only the aromatic region is shown for clarity. The HMBC spectrum displayed here (dark blue) is the sum of two separate HMBC spectra, one optimized for 5 Hz and the other for 10 Hz ^{1}H-^{13}C scalar couplings. The solution-state ^{1}H NMR spectrum and the solid-state ^{13}C photo-CIDNP spectrum are shown as a guide for the eye. The background of the 2D plot has been highlighted with different colors corresponding to different parts of the CarboPol structure (red: donor, yellow: chromophore, blue: acceptor, black: end group).

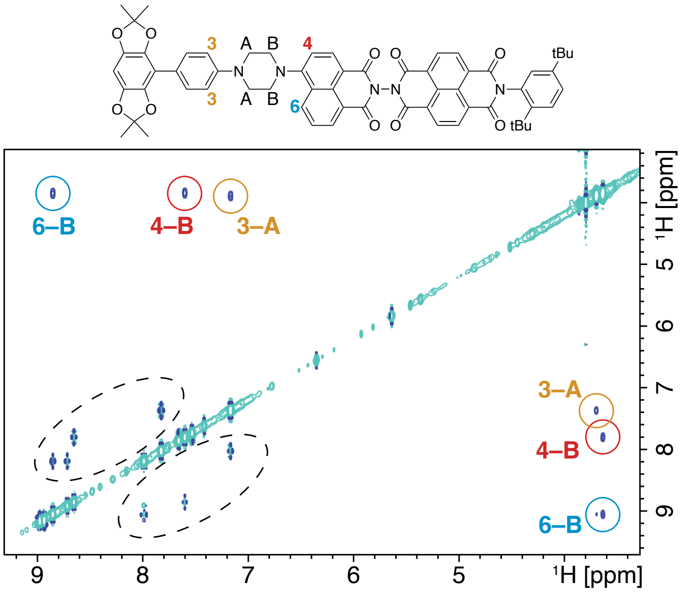

Figure S6. ^{1}H-^{1}H NOESY spectrum of 5 mM CarboPol in acetone-d_6 (500 MHz ^{1}H Larmor frequency). Cross peaks highlighted with colored circles denote various NOE contacts within the methylene groups (labelled A and B) and protons 3, 4 and 6 in the CarboPol structure. Cross peaks within the dashed circles are artifacts.

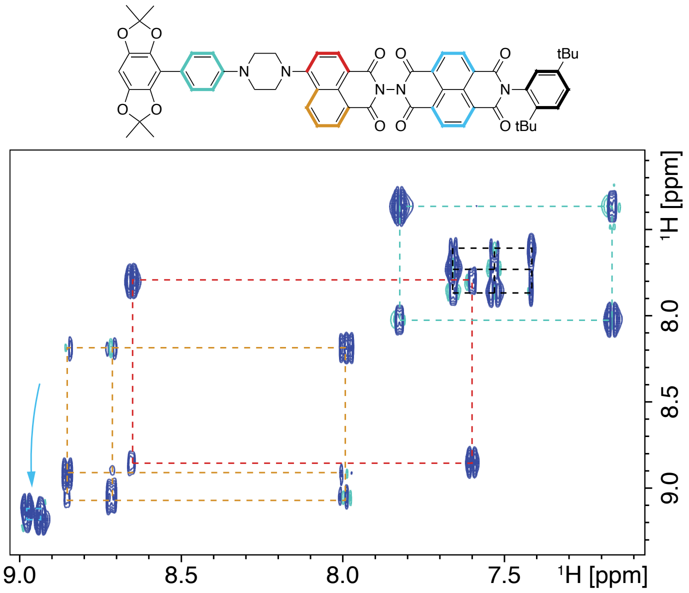

Figure S7. ^1H-^1H TOCSY spectrum of 5 mM CarboPol in acetone-d_6 (500 MHz ^1H Larmor frequency). The various spin systems in the structure are indicated with different colors. The light blue arrow in the 2D plot is used to further highlight the acceptor spin system at the bottom left of the spectrum.

3) Experiments on 15 mM CarboPol samples

To calculate the ^{13}C photo-CIDNP enhancement, $\varepsilon = I_{on}/I_{off}$, the signal intensity without the laser (I_{off}) is required. For the 1.5 mM sample, CarboPol aromatic signals at 138 ppm could not be detected without the laser. In the attempt to measure them, two additional CarboPol samples in OTP were prepared with 10× higher concentrations, i.e., 15 mM. First, for both 15 mM samples and for the 1.5 mM sample used to collect the spectra in Fig. 2 of the main text, a laser-off spectrum was acquired at 100 K with a 2 h relaxation delay (Fig S8). The intensity of the OTP signal is similar in all the three spectra, indicating that the absolute amount of frozen solution in the samples is comparable. Even with the 10× higher concentration, no CarboPol signals can be observed here after four scans.

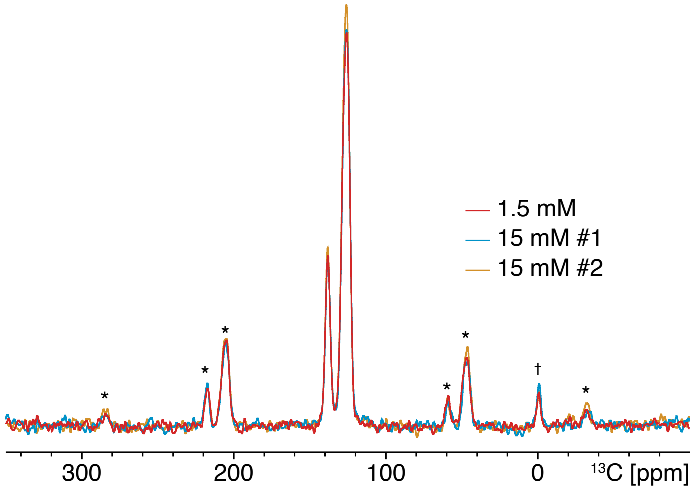

Figure S8. ^{13}C NMR laser-off spectra of three different frozen solutions of CarboPol in OTP (9.4 T, 8 kHz MAS, 100 K). The concentration of CarboPol for each sample is indicated inset. All spectra have been acquired with 4 scans (2 h interscan delay, 90° excitation pulse). The asterisks indicate spinning sidebands while the dagger denotes the silicone plug signals.

We measured the laser-off spectra for the 15 mM samples using the same parameters as Fig. 2 of the main text (1.8 s interscan delay, 68° excitation pulse and 30,000 scans, Fig. S9). For the higher concentration samples (15 mM), the faster-relaxing CarboPol methyl signals are now visible at 30 ppm after 30,000 scans. Since the 138 ppm CarboPol signals overlap with OTP, we subtracted the OTP signal from the 15 mM laser-off spectra using the laser-off spectrum of the 1.5 mM sample (Fig. S10). Given the slightly different OTP signal intensity among the samples, the 1.5 mM spectrum was rescaled according to the intensity of the OTP signal at 126 ppm prior to each subtraction.

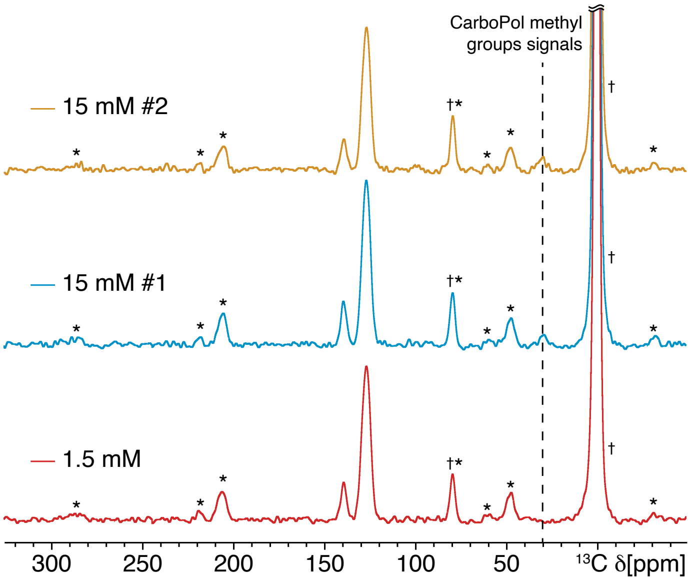

Figure S9. ^{13}C NMR laser-off spectra of three different frozen solutions of CarboPol in OTP (9.4 T, 8 kHz MAS, 100 K). The CarboPol concentration for each spectrum is indicated inset. The interscan delay was set equal to the polarization build-up time under laser irradiation (T_b = 1.8 s) and an excitation pulse of 68° was used to acquire the signal. All spectra were acquired with 30,000 scans. The asterisks indicate spinning sidebands while the daggers denote the silicone plug signals. The dashed line indicates the position of the ^{13}C signal of the various methyl groups in CarboPol, visible only in the spectra of the two concentrated samples. For CarboPol, only the methyl groups signal is detected with sufficient sensitivity because of the faster longitudinal relaxation.

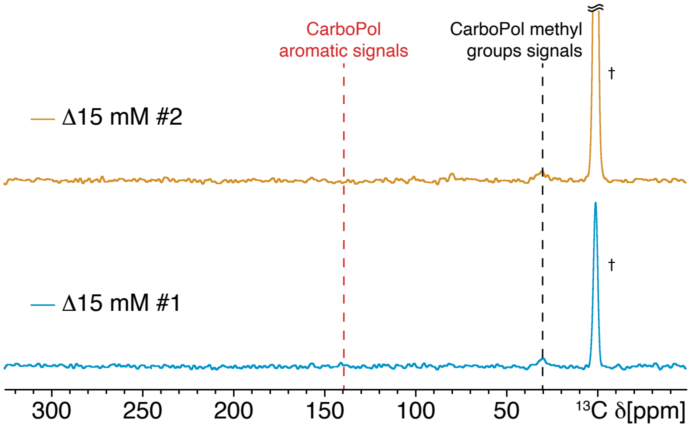

Figure S10. ^{13}C NMR difference spectra obtained by subtracting the laser-off spectrum of the 1.5 mM sample from each of the two 15 mM samples. In both cases, the 1.5 mM spectrum was rescaled according to the intensity of the OTP signal at 126 ppm. The daggers denote the silicone plug signals while the black dashed line indicates the position of the methyl groups signal of CarboPol. The red dashed line is placed at 138 ppm, where the CarboPol aromatic signals are expected. No aromatic resonance is observed above the noise level.

Based on the noise level in the difference spectra, we can determine for both cases a lower bound on the enhancement for the 1.5 mM sample with a 1.8 s recycle delay:

$$|\varepsilon_{13\mathrm{C}}| > \frac{I_{\mathrm{on}}^{(1.5\ \mathrm{mM})}}{N_{\mathrm{off}}^{(\Delta 15\ \mathrm{mM})}} \times \frac{15 - 1.5}{1.5} = \frac{I_{\mathrm{on}}^{(1.5\ \mathrm{mM})}}{N_{\mathrm{off}}^{(15\ \mathrm{mM})}} \times 9 \approx 2{,}300$$

where the factor of 9 comes from the subtraction of the 1.5 mM spectrum from each of the 15 mM spectrum.

4) Additional solid-state NMR experiments

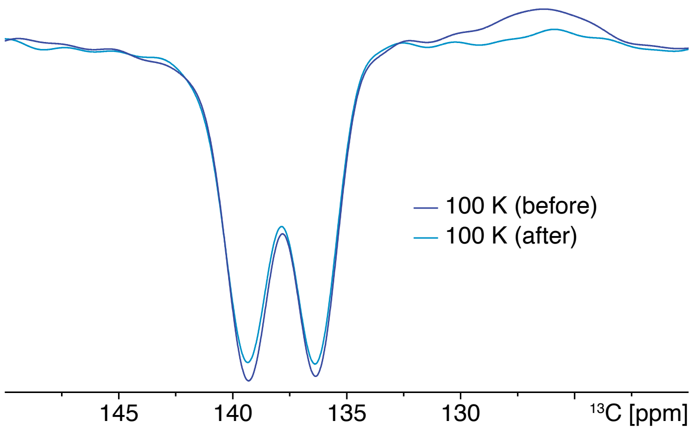

Figure S11. ^{13}C NMR spectra of a 1.5 mM frozen solution of CarboPol in OTP (9.4 T, 8 kHz MAS) with continuous laser irradiation (1.8 s recycle delay, 2,000 scans) acquired before and after the temperature dependence study in Fig. 5 of the main text. The photo-CIDNP-enhanced CarboPol signal in the two spectra are comparable, suggesting only minimal or no degradation of the sample under irradiation with light at temperatures up to 220 K.

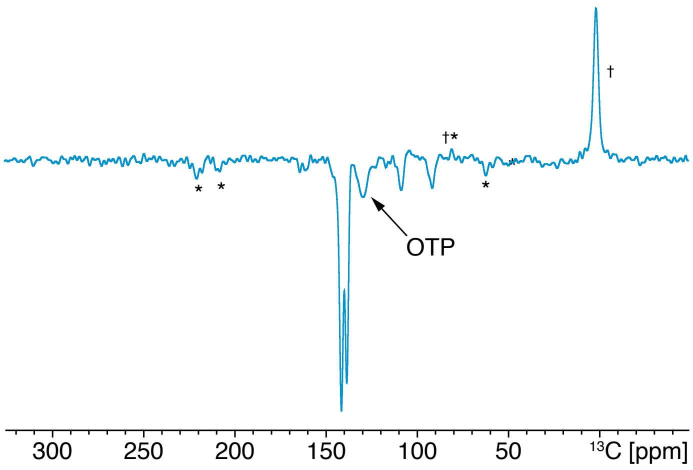

Figure S12. ^{13}C NMR spectrum of a 1.5 mM frozen solution of CarboPol in OTP (9.4 T, 8 kHz MAS, 100 K) with 450 nm laser irradiation (20.48 s interscan delay, 128 scans, 90° excitation pulse). The asterisks indicate spinning sidebands while the daggers denote the silicone plug signal. At this interscan delay, the OTP signal becomes negative because of relayed polarization from CarboPol via ^{13}C–^{13}C spin diffusion.

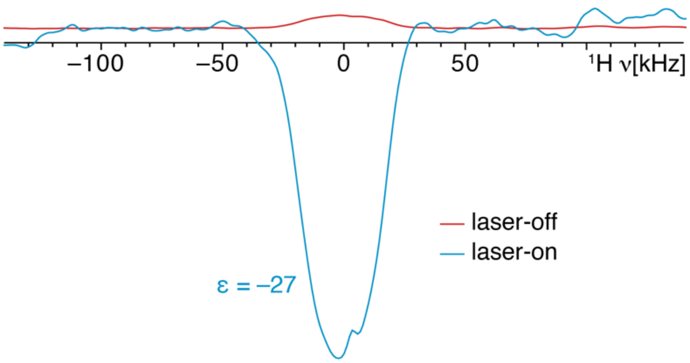

Figure S13. ^{1}H NMR spectra of a 1 mM frozen solution of CarboPol in OTP (85 K, static solid, 0.3 T, RF carrier at 12.769 MHz) without (red, 3,000 scans) and with (blue, 50 scans) continuous laser irradiation at 450 nm. A short (15 μs) solid echo pulse sequence was applied prior to signal acquisition and the recycle delay was 20 s. Note that the greater noise level in the laser-on spectrum is due to 60 times fewer scans being acquired.

5) Electron–electron interaction in CarboPol

Samples of CarboPol were prepared in toluene or butyronitrile with optical densities (O.D.) = 0.5 – 0.7 at 415 nm for measurements at 295 K and 85 K, respectively. The samples were placed in borosilicate rectangular sample cuvettes (2×4 mm, 0.5 mm wall), then subjected to three freeze-pump-thaw cycles on a vacuum line (10^{-4} Torr) and sealed with a hydrogen torch. The cuvettes were placed in a nitrogen-cooled cryostat (STVP-100 Janis) positioned between the poles of a Walker Scientific HV-4W electromagnet powered by a Walker Magnion HS-735 power supply. The field strength was measured by a Lakeshore 475 DSP Gaussmeter with a Hall effect probe. The samples were pumped using 415 nm, 5 ns laser pulses at a 1 kHz repetition rate from an NT242 Ekspla laser. The white light probe pulses were generated at 2 kHz repetition rate using a Leukos white light supercontinuum laser. After passing through the sample, the probe light was filtered using a 10 nm bandpass filter centered at 500 nm and directed into a photomultiplier tube (Hamamatsu H9307-03). The signal was then monitored using a lock-in amplifier (Stanford Research Systems SR830 DSP) with a 1 kHz reference signal from the pump laser. For measurements at 295K, changes in the intensity of the triplet–triplet absorption of ^{3*}NDI produced by recombination of BDX$^{•+}$-ANI-NDI$^{•-}$ were monitored at a 1 μs pump–probe delay, while the magnetic field was swept from 0 to 40 mT in 0.5 mT steps. At 85 K, the changes in the BDX$^{•+}$-ANI-NDI$^{•-}$ transient absorption were monitored at a 10 μs pump–probe delay, while the magnetic field was swept from 0 to 10 mT in 0.1 mT steps. The transient absorption changes at 295 K exhibited a resonance at $B_{1/2} = 2J = 20$ mT (≈ 560 MHz), while $B_{1/2} =$ 2 mT (≈ 56 MHz) was observed at 85 K.

6) Synthetic procedures

General information. Unless otherwise noted, reagents were purchased from Sigma-Aldrich, Inc. ^{1}H and ^{13}C NMR spectra were acquired using a 500 MHz Bruker Avance III NMR spectrometer equipped with DCH CryoProbe. Mass spectra were collected using a Bruker RapiFlex MALDI-TOF. Purification was performed using silica gel from Sorbent Technologies.

Scheme S1 outlines the synthesis of compound **1** (CarboPol). The synthesis of 1-benzyl-4-(4-(2,2,6,6-tetramethylbenzo[1,2-d:4,5-d']bis([1,3]dioxole)-4-yl)phenyl)piperazine (BDX-Ph-piperazine-Bn, **2**), and the corresponding deprotected piperazine (BDX-Ph-piperazine, **3**), have been described previously.[1] Compound **4** was also prepared previously.[2]

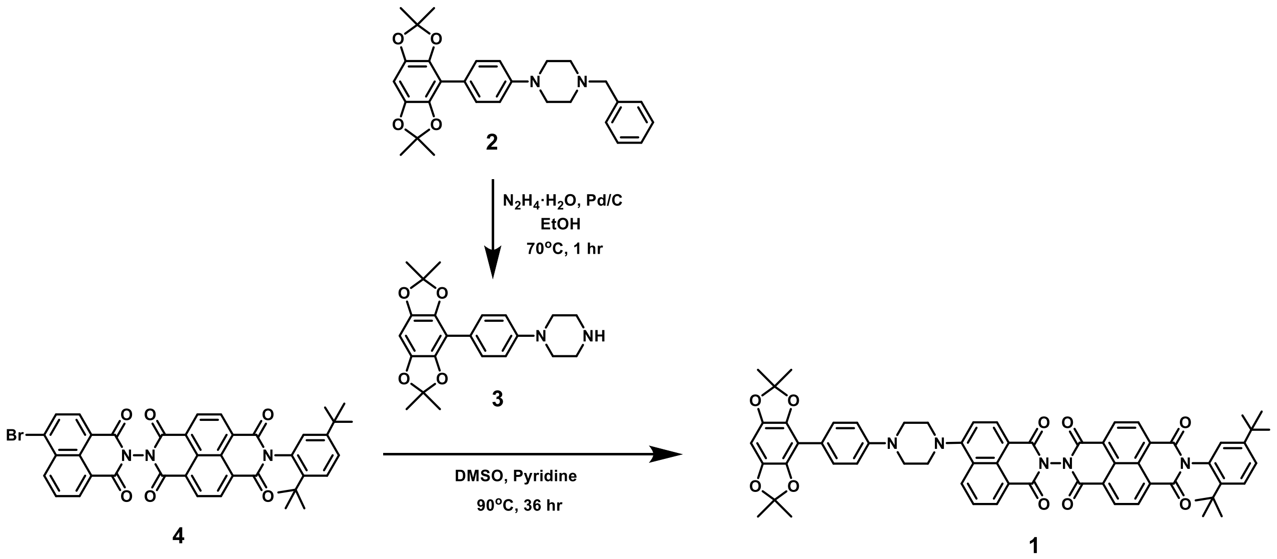

Scheme S1. Synthesis of compound **1** (CarboPol).

Compound 1. A flame-dried round-bottom flask equipped with a magnetic stir bar was charged with **3** (62 mg, 0.16 mmol, 2.0 equiv.) and **4** (58 mg, 0.08 mmol, 1.0 equiv.), sealed with a rubber septum, and then subjected to three pump-purge cycles with N_2. A degassed mixture of 2 mL anhydrous DMSO and 1 mL anhydrous pyridine was added through the septum, and the mixture was then heated to 90 °C for 36 h under N_2. The reaction mixture was cooled to room temperature, diluted with water (10 mL), extracted with DCM (3×10 mL), dried over Na_2SO_4, concentrated by rotary evaporation, and purified by column chromatography on silica gel (eluent: DCM/Acetone (20/1, v/v)) to yield a brownish yellow solid as the final product **1** (26 mg, 25 μmol, 32%). ^{1}H NMR (500 MHz, $CDCl_3$): δ = 8.86–8.92 (dd, 4H), 8.69–8.73 (dd, 1H), 8.63–8.66 (dd, 1H), 8.59–8.63 (dd, 1H), 7.78–7.85 (m, 3H), 7.60–7.64 (m, 1H), 7.48–7.52 (m, 1H), 7.32–7.37 (m, 1H), 7.06–7.11 (m, 2H), 7.03–7.05 (d, 1H), 6.29 (s, 1H), 3.55–3.63 (m, 4H), 3.48–3.55 (m, 4H), 1.70 (s, 12H), 1.35 (s, 9H), 1.29 (s, 9H). ^{13}C NMR (126 MHz, $CDCl_3$): δ = 163.70, 161.27, 161.17, 160.67, 160.57, 159.88, 157.09, 150.44, 149.92, 143.75, 140.68, 137.88, 133.97, 132.53, 132.05, 131.96, 131.65, 131.48, 130.57, 129.98, 129.03, 127.71, 127.58, 127.51, 127.48, 126.73, 126.62, 126.01, 124.04, 122.64, 117.54, 115.98, 115.78, 115.28, 108.23, 91.14, 53.12, 49.10, 35.61, 34.32, 31.76, 31.22, 29.71, 25.76. MS (MALDI-TOF) m/z: calculated for $C_{62}H_{55}N_5O_{10}$, $[M]^+$ 1029.39; found 1028.97.

References

1. Carmieli, R.; Mi, Q.; Ricks, A. B.; Giacobbe, E. M.; Mickley, S. M.; Wasielewski, M. R., Direct measurement of photoinduced charge separation distances in donor–acceptor systems for artificial photosynthesis using oop-eseem. *J. Am. Chem. Soc.* **2009,** *131*, 8372-8373.

2. Horwitz, N. E.; Phelan, B. T.; Nelson, J. N.; Mauck, C. M.; Krzyaniak, M. D.; Wasielewski, M. R., Spin polarization transfer from a photogenerated radical ion pair to a stable radical controlled by charge recombination. *J. Phys. Chem. A* **2017,** *121*, 4455-4463.
